# The IL-3, IL-5, and GM-CSF common receptor beta chain mediates oncogenic activity of FLT3-ITD-positive AML

**DOI:** 10.1038/s41375-021-01462-4

**Published:** 2021-11-08

**Authors:** Anne Charlet, Max Kappenstein, Philip Keye, Kathrin Kläsener, Cornelia Endres, Teresa Poggio, Sivahari P. Gorantla, Stefanie Kreutmair, Jana Sänger, Anna L. Illert, Cornelius Miething, Michael Reth, Justus Duyster, Christoph Rummelt, Nikolas von Bubnoff

**Affiliations:** 1 Department of Hematology, Oncology and Stem Cell Transplantation, Medical Center, Faculty of Medicine, University of Freiburg, Freiburg, Germany; 2 Eye Center, Medical Center, Faculty of Medicine, University of Freiburg, Freiburg, Germany; 3 Signalling Research Centres BIOSS and CIBSS, University of Freiburg, Freiburg, Germany; 4 Institute for Biology III, Faculty of Biology, University of Freiburg, Freiburg, Germany; 5 German Cancer Research Center (DKFZ), Heidelberg, Germany; 6 Faculty of Biology, Albert Ludwigs University of Freiburg, 79106 Freiburg, Germany; 7 Department of Hematology and Oncology, Medical Center, University of Schleswig-Holstein, Campus Lübeck, Lübeck, Germany; 8 Institute of Allergy Research, Helmholtz Zentrum Munich, Munich, Germany; 9 German Cancer Consortium (DKTK), partner site Freiburg, Freiburg, Germany

**Keywords:** Acute myeloid leukaemia, Translational research, Acute myeloid leukaemia, Oncogenesis

## Abstract

FLT3-ITD is the most predominant mutation in AML being expressed in about one-third of AML patients and is associated with a poor prognosis. Efforts to better understand FLT3-ITD downstream signaling to possibly improve therapy response are needed. We have previously described FLT3-ITD-dependent phosphorylation of CSF2RB, the common receptor beta chain of IL-3, IL-5, and GM-CSF, and therefore examined its significance for FLT3-ITD-dependent oncogenic signaling and transformation. We discovered that FLT3-ITD directly binds to CSF2RB in AML cell lines and blasts isolated from AML patients. A knockdown of CSF2RB in FLT3-ITD positive AML cell lines as well as in a xenograft model decreased STAT5 phosphorylation, attenuated cell proliferation, and sensitized to FLT3 inhibition. Bone marrow from CSF2RB-deficient mice transfected with FLT3-ITD displayed decreased colony formation capacity and delayed disease onset together with increased survival upon transplantation into lethally irradiated mice. FLT3-ITD-dependent CSF2RB phosphorylation required phosphorylation of the FLT3 juxtamembrane domain at tyrosines 589 or 591, whereas the ITD insertion site and sequence were of no relevance. Our results demonstrate that CSF2RB participates in FLT3-ITD-dependent oncogenic signaling and transformation in vitro and in vivo. Thus, CSF2RB constitutes a rational treatment target in FLT3-ITD-positive AML.

## Introduction

The internal tandem duplication (ITD) mutation of the class III receptor tyrosine kinase FMS-like Tyrosine Kinase 3 (FLT3) is found in 25% of all AML cases and is associated with a dismal prognosis [1]. Despite recent advances with combinations of standard chemotherapy and specific FLT3 tyrosine kinase inhibitors (TKIs), the only cure for patients diagnosed with a high FLT3-ITD allelic ratio AML is allogeneic stem cell transplantation [2]. The ITD mutation is predominantly located within the juxtamembrane domain (JMD) of FLT3 and leads to constitutive activation of the receptor and to downstream signaling events in the absence of ligand [3]. It has been demonstrated that FLT3-ITD shares activation of PI3K-AKT and MAPK (ERK) with ligand-stimulated FLT3, but in addition strongly activates STAT5 [3–5]. The mechanism of STAT5 activation by FLT3-ITD is unknown. Direct activation has been proposed [6] as well as activation via signaling intermediates such as SRC, GRB2, BTK, or SYK [7–11].

The FLT3 TKIs midostaurin and gilteritinib were recently approved for the treatment of FLT3-mutated AML [12, 13]. The main limitation of TKI treatment is the rapid development of resistance [14–16]. It is known that IL-3, IL-5, and GM-CSF-dependent STAT5 phosphorylation requires phosphorylation of JAK2 and CSF2RB, the common beta chain of IL-3, IL-5, and GM-CSF receptors (CD131, IL3RB) [17, 18]. We have recently demonstrated that activating JAK-family mutations mediated resistance to FLT3 inhibitors [19]. In cells expressing FLT3-ITD, JAK mutations reestablished phosphorylation of CSF2RB and STAT5 in the presence of FLT3 inhibitor. Strikingly, in FLT3 TKI-naive JAK wildtype (WT) cells, CSF2RB was phosphorylated in a JAK2 independent, but FLT3-ITD dependent fashion that disappeared upon FLT3 inhibition, suggesting that CSF2RB participates in FLT3-ITD downstream signal activation. In this work, we investigated the significance of CSF2RB in FLT3-ITD positive AML. We here demonstrate that CSF2RB binds FLT3-ITD and participates in FLT3 dependent STAT5 activation and oncogenic transformation in vitro and in vivo.

## Materials and methods

### Cell culture and viral transduction

Ba/F3, MOLM-13, MV4–11, and THP-I were cultured in Roswell Park Memorial Institute 1640 supplemented with 10% fetal bovine serum (FBS) and penicillin/streptomycin, for Ba/F3 additional 2 ng/ml IL-3. OCI-AML3 were cultured in alpha-MEM with 20% FBS and penicillin/streptomycin. Phoenix Eco (Gary Nolan, Stanford, CA) were cultured in Dulbecco’s Modified Eagle Medium with 10% FBS and periodically selected with hygromycin 300 mg/ml and diphtheria toxin 2 µg/ml. MV4–11, MOLM-13, and OCI-AML3 cells were kindly provided by M. Lübbert (Freiburg, Germany). Cell lines were generated via retroviral transduction via Phoenix Eco cells. Human cell lines expressed the murine ecotropic receptor Slc7a1. All Cells were incubated at 37 °C with 5% CO_2_. Midostaurin was a kind gift from Novartis (Basel, Switzerland). Sorafenib was purchased from American Chemicals Custom Corporation (San Diego, USA). Gilteritinib was purchased from Selleckchem (Pittsburg, USA).

### DNA constructs

Human CSF2RB cDNA was kindly provided by M. Martinez-Moczygemba (Houston, USA) and subcloned into pMSCV. Human FLT3 constructs were expressed using puromycin-IRES-GFP (PIG). FLT3 and FLT3-ITD 598/599 [12] were kindly provided by F. Heidel (Greifswald, Germany). FLT3-ITD (murine-human hybrid) pMSCV-IRES-GFP (MIG) and FLT3 constructs were kindly provided by H. Serve (Frankfurt, Germany). The shRNA for CSF2RB was designed using SplashRNA algorithm [20] (for murine knockdown targeting both loci) and was subcloned into pLMN for constitutive knockdown and into TREBAV for an inducible knockdown in combination with pMSCV-RIEP [21]. Plasmids pcDNA3.1 zeo (-) and pGEX4T1 were used for GST-pulldown. All constructs derived from site-directed mutagenesis and PCR were verified by automated sequence analysis.

### Western blot analysis and immunoprecipitation

Immunoblotting and immunoprecipitation were performed as previously described [22]. Following antibodies were used, sorted by manufacturer: FLT3, CSF2RB, AKT, tubulin, vinculin, anti-GST sourced from Santa Cruz (Heidelberg, Germany). Phoshpo-FLT3 589/591, β-Actin, phospho-AKT, ERK/p44/42 MAPK, phospho-ERK/phospho-p44/42 MAPK, STAT5, phospho-STAT5, and horseradish peroxidase-linked antibodies were obtained from Cell Signaling (Frankfurt, Germany). Phospho-tyrosine antibodies were purchased from Merck and BD Transduction Laboratories (Allschwil, Switzerland). Phospho-FLT3 Y599 antibody was purchased from Thermo Scientific (Dreieich, Germany). Anti-FLAG beads (ANTI-FLAG M2 Affinity Gel) were obtained from Sigma Aldrich (Taufkirchen, Germany) and Protein A agarose beads from GE Healthcare (Freiburg, Germany).

### GST-pulldown assay

Sequences of FLT3 and CSF2RB were cloned into pGEX-4T-1. GST fusion proteins were expressed in *Escherichia coli* BL21, purified, and immobilized on glutathione-sepharose beads (Glutathione Sepharose 4B, GE Healthcare, Freiburg, Germany). Cytoplasmic fragments of FLT3 and CSF2RB used for T7 in vitro translation (TNT Quick Coupled Transcription/Translation System, Promega, Walldorf, Germany) were cloned into pcDNA3.1. GST-tagged peptides captured by glutathione-sepharose beads were incubated with in vitro translated target protein overnight at 4 °C. Interacting complexes were recovered by collecting washed beads. Analysis and detection of retained target proteins were performed by sodium dodecyl sulfate–polyacrylamide gel electrophoresis and western blotting.

### In situ proximity ligation assay

Oligo-coupled primary antibodies (1-PLA) were used to restrict the detection of a nanoscale protein:protein interaction to a 10–40 nm range as previously described [23]. As primary antibodies anti-human FLT3 (clone A2F10) and anti-human CSF2RB (clone 1 C,1 eBioscience, Frankfurt, Germany) were used. Patient sample collection and analyses were approved by the institutional review board of the University of Freiburg Medical Center. Peripheral blood samples from patients with FLT3-mutated AML and a control AML patient were taken after informed consent. MOLM-13, MV4–11, OCI-AML3, or human Ficoll separated blast cells from AML patients were settled on polytetrafluoroethylene slides (Thermo Scientific, Dreieich, Germany) and fixed with 4% paraformaldehyde. To inhibit FLT3 kinase activity, midostaurin [100 nM] was added for 60 min. After the PLA amplification reaction, cells were mounted and nuclei were stained with 4′,6-diamidino-2-phenylindole (DAPI) Fluoromount-G (Biozol, Eching, Germany). Microscopy images were acquired with a Zeiss 780 Meta confocal microscope (Carl Zeiss, Jena, Germany). For each experiment, a minimum of 1000 cells or 500 patient cells from several images were analyzed (ImageJ and CellProfiler 3.0.0) and the mean PLA signal count per cell was calculated. Statistical significance was computed via an unpaired nonparametric Mann–Whitney *U* test.

### Proliferation assays

Cell viability was measured using an MTS-based method by absorption of formazan at 490 nm (CellTiter 96; Promega, Madison, USA). Cells were plated at a density of 6000 or 10000 cells per well in a 96-well plate and treated with inhibitors at indicated concentrations. Measurements were taken in triplicates after 48 or 72 h.

### Animal experiments

All animal experiments were conducted in accordance with national and local ethical regulations and were approved by the Regierungspräsidium Freiburg, Germany (Protocol number: G-16/164). Mice used for experiments were between 8 and 12 weeks old.

### Xenografts and in vivo bioluminescence imaging (BLI)

For the leukemia transplantation model sublethally irradiated C;129S4-Rag2^tm1.1Flv Il2rgtm1.1Flv^/J knockout mice (JAX stock #014593) [24] were injected with 1 × 10^5^ MOLM-13 cells expressing control shRNA or CSF2RB shRNA and luciferase into their tail veins. BLI measurements were performed using the IVIS200 imaging system (Xenogen) after intraperitoneal injection of luciferin (150 mg/kg; BioSynth, Staat, Switzerland). Data were quantified with Living Image Software (Xenogen). For subcutaneous tumor model C;129S4-Rag2^tm1.1Flv Il2rgtm1.1Flv^/J knockout mice were subcutaneously injected with 5 × 10^6^ MOLM-13 cells expressing control shRNA or CSF2RB shRNA into their right flanks. Tumor size was measured by a caliper and calculated as described here [25]. Tumor weight was measured after sacrificing the mice on day 17. Staining was performed using antibodies against phospho-STAT5 (see above) or CSF2RB (Abcam, Cambridge, UK).

### Methylcellulose assay and bone marrow transplantation

C57BL/6 J B6.129S1-Csf2rb2^tm1Cgb^ Csf2rb1^tm1Clsc^/J double knockout (JAX stock #005963, RRID:IMSR_JAX:005963, Bar Harbor, ME, USA) [26] or double WT donor mice (littermates) were treated with 5-FU 4 days before bone marrow (BM) harvest. For methylcellulose assays, BM was cultured and transfected with PIG hFLT3-ITD 598/599 construct or PIG empty vector. Transduced cells (GFP^+^) were isolated using an automated cell sorter (BD Biosciences, Heidelberg, Germany), seeded into methylcellulose (STEMCELL Technologies, Cologne, Germany), and cultured without or in the presence of cytokines (PeproTech, Hamburg, Germany). Colonies were counted on day 7. For transplantation, BM was transduced with pMIG FLT3-ITD, resulting in 7.5% GFP+ cells in both samples after equalization. Transplantation was performed via tail vein injection (200,000 cells/mouse) in lethally irradiated (2 × 4.5 Gy) C57BL/6 J-recipient mice.

### Statistical analysis and quantification

Statistical analysis was performed using GraphPad 5.0 (La Jolla, USA). Data are presented as mean ± SEM and comparisons were calculated by Student’s *t* test (two-sided, unpaired) unless otherwise indicated. All experiments were repeated at least three times in triplicates unless otherwise indicated. Results were considered statistically significant for *p* < 0.05.

## Results

### CSF2RB interacts with FLT3 and is phosphorylated in an FLT3-ITD-dependent manner

**Fig. 1:**
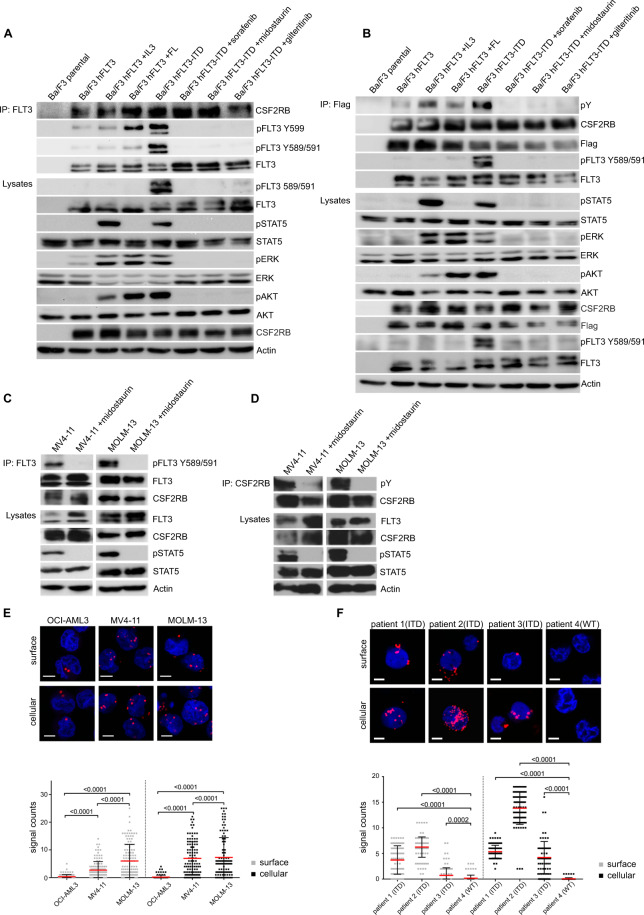
CSF2RB interacts with FLT3. **A**, **B** Ba/F3 cells expressing CSF2RB-Flag and either FLT3 or FLT3-ITD were serum-deprived for 5 h with addition of 2 ng/ml IL-3 (5 min), 50 ng/ml FLT-ligand (5 min), 100 nM midostaurin (1.5 h), 300 nM sorafenib (1,5 h) or 100 nM gilteritinib (1,5 h) as indicated. Co-immunoprecipitations were performed using FLT3-antibody (**A**) or anti-flag beads capturing CSF2RB (**B**). Immunoprecipitates and whole-cell lysates were subjected to SDS–PAGE and western blot analysis using indicated antibodies. **C**, **D** FLT3-ITD-positive AML cell lines MV4–11 and MOLM-13 were serum-deprived for 4 h and additionally treated with 100 nM midostaurin for 1.5 h as indicated. Co-immunoprecipitations were performed using FLT3-antibody (**C**) or CSF2RB-antibody (**D**). Immunoprecipitates and whole-cell lysates were subjected to SDS–PAGE and western blot analysis using indicated antibodies. **E** Proximity ligation assay (1-PLA) was performed using oligo-coupled primary antibodies against FLT3 and CSF2RB. FLT3 expressing OCI-AML3 cells and FLT3-ITD expressing MV4–11 and MOLM-13 cells were fixed (surface) or fixed and permeabilized with 0.5% saponine (cellular) prior to PLA reaction. Red dots indicate the occurrence of a close FLT3: CSF2RB proximity. Nuclei were counterstained with DAPI. Representative images are shown (Zeiss 780 Meta confocal microscope; Objective NA 1.4), scale bar = 10 µm. Quantification of the PLA signals is shown as signals per cell. **F** Blast cells from three FLT3-ITD positive AML patients and one FLT3-ITD negative AML patient (patient 4) were isolated from peripheral blood using Ficoll density gradient. Cells were fixed (surface) or fixed and permeabilized (cellular) and PLA was performed (as described in E), *p* ≤ 0.0002.

We have previously shown that expression of FLT3-ITD leads to phosphorylation of CSF2RB [19]. First, we asked whether FLT3-ITD binds to CSF2RB and is necessary and sufficient for CSF2RB phosphorylation. Both, FLT3 and FLT3-ITD co-immunoprecipitated overexpressed (Fig. 1A) and endogenous CSF2RB (Suppl. Fig. 1A) in Ba/F3 cells. However, phosphorylation of CSF2RB and downstream activation of STAT5 only occurred in the presence of FLT3-ITD (Fig. 1B). Phosphorylation of CSF2RB and activation of STAT5 was suppressed upon inhibition of FLT3 by midostaurin, sorafenib, or gilteritinib, whereas the interaction between CSF2RB and FLT3 remained unaltered (Fig. 1B). Of note, treatment of Ba/F3 cells expressing FLT3 with FLT-ligand resulted in phosphorylation of FLT3 at tyrosine 599 [27] and not, as in FLT3-ITD transfected cells, additionally at tyrosines 589 and 591 (Fig. 1A). Also, FLT-ligand treatment led to phosphorylation of ERK and AKT, but not CSF2RB and STAT5 (Fig. 1B). We confirmed the CSF2RB/FLT3-ITD interaction in the FLT3-ITD positive human primary leukemic cell lines MOLM-13 and MV4–11 (Fig. 1C). Again, phosphorylation of CSF2RB and activation of STAT5 was suppressed by midostaurin (Fig. 1D). In situ proximity ligation assay (PLA) experiments confirmed the CSF2RB/FLT3-ITD interaction in MOLM-13 and MV4–11 cells even on a single-molecule level, suggesting a direct interaction [28]. In FLT3 expressing human OCI-AML3 cells, considerably fewer PLA signals were counted (Fig. 1E). The PLA dot counts were not altered upon midostaurin treatment, demonstrating that FLT3-ITD kinase activity is not required for the CSF2RB/FLT3-ITD interaction (Suppl. Fig. 2A, B). A PLA study of Ficoll-isolated blast cells from three FLT3-ITD-positive AML patients and one FLT3-ITD-negative AML patient (patient 4) confirmed the interaction in primary AML blasts (Fig. 1F). Taken together, these data demonstrate that FLT3 forms a complex with CSF2RB and that CSF2RB is phosphorylated in an FLT3-ITD-dependent fashion.

### CSF2RB is essential for FLT3-ITD induced proliferation

**Fig. 2:**
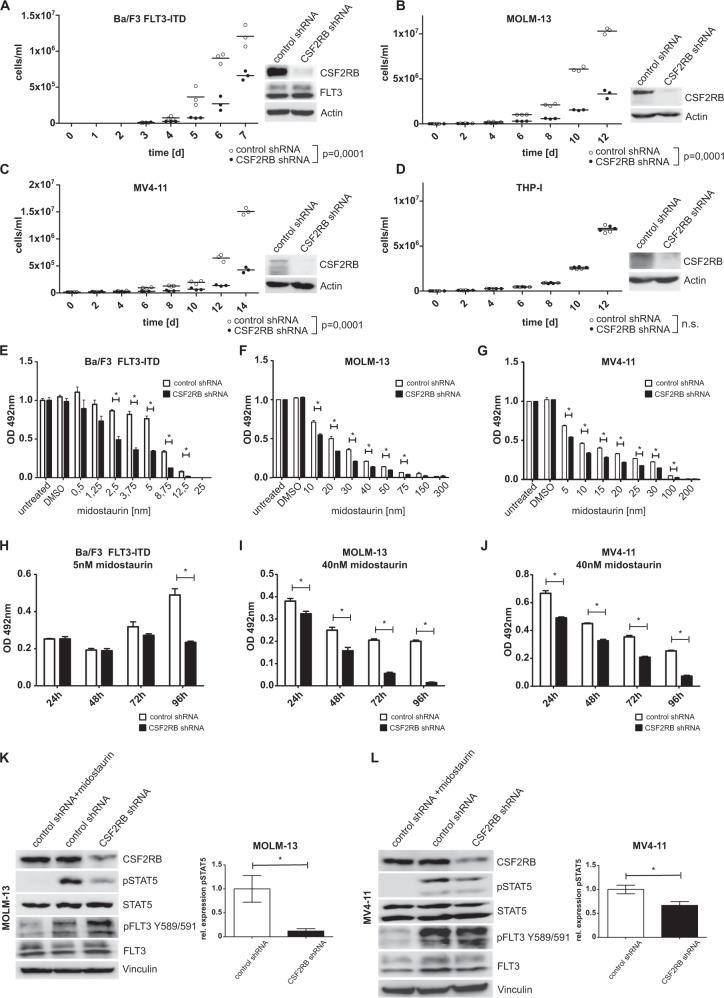
CSF2RB knockdown impairs FLT3-ITD-induced cell growth, sensitizes cells to FLT3 inhibition, and decreases phosphorylation of STAT5. **A**–**J** Ba/F3 cells transduced with FLT3-ITD, FLT3-ITD-positive AML cell lines MOLM-13 and MV4–11, as well as FLT3 expressing cell line THP-I, were transduced with constructs bearing inducible CSF2RB shRNA or control shRNA, respectively, and cultured in the presence of doxycycline for at least 48 h prior to and during experiments. **A**–**D** Proliferation of control shRNA vs. CSF2RB shRNA-expressing cells was determined by counting viable cells after trypan blue staining in Neubauer counting chamber at indicated time points. Cells were seeded in triplicates in a density of 1 × 10^4^cells/ml at day 0. Fresh medium was added regularly to maintain optimal cell densities over time. Cell lysates were subjected to SDS–PAGE and western blot analysis using indicated antibodies to confirm knockdown. **E**–**G** Cell viability was determined using MTS assay. Cells were seeded in a density of 6000 cells per well in 96-well plates and cultured in the presence of midostaurin at the indicated concentrations. Proliferation was measured in triplicates as formazan absorption after 72 h at 490 nM. **H**–**J** Cells were seeded in a density of 6000 cells per well in 96-well plates and cultured in the presence of midostaurin at the indicated concentrations. Cell viability was measured daily by formazan absorption at 490 nm. **K**, **L** Cells were serum-deprived for 8 h, with or without the addition of 25 nM midostaurin. Whole-cell lysates were subjected to SDS–PAGE and western blot analysis using the indicated antibodies. Quantification of pSTAT5 relative to total STAT5 was calculated from three biologically independent replicates.

Activated CSF2RB leads to STAT5 phosphorylation [17, 18], the key mediator of oncogenic FLT3-ITD signaling [4]. As we observed that FLT3-ITD induces phosphorylation of CSF2RB, we reasoned that CSF2RB is required for the oncogenic transformation of FLT3-ITD positive cells. We thus performed shRNA-induced knockdown of CSF2RB in FLT3-ITD expressing Ba/F3 cells, FLT3-ITD-positive human cell lines MOLM-13 and MV4–11, and FLT3 expressing human THP-I cells. CSF2RB knockdown impaired proliferation in all FLT3-ITD expressing cell lines, but not in FLT3 expressing THP-I cells (Fig. 2A–D). Furthermore, CSF2RB knockdown sensitized FLT3-ITD expressing Ba/F3, MOLM-13, and MV4–11 cells to growth inhibition induced by the FLT3 inhibitor midostaurin (Fig. 2E–G) and increased the proportion of apoptotic cells in MOLM-13 and MV4–11 cells (Suppl. Fig. 2C). Moreover, midostaurin at a concentration of 5 nM in Ba/F3 cells expressing FLT3-ITD and of 40 nM in MOLM-13 and MV4–11 synergized with CSF2RB knockdown to block the outgrowth of these cells (Fig. 2H–J). Usage of the more FLT3 specific inhibitor gilteritinib confirmed these results in FLT3-ITD expressing Ba/F3 and MV4–11 cells (Suppl. Fig. 3A–D). CSF2RB knockdown decreased the phosphorylation of STAT5 in MOLM-13 and MV4–11 cells (Fig. 2K–L). These observations demonstrate the biologic relevance of CSF2RB in mediating FLT3-ITD’s oncogenic potential in vitro.

### CSF2RB mediates FLT3-ITD-dependent oncogenic activity in vivo

**Fig. 3:**
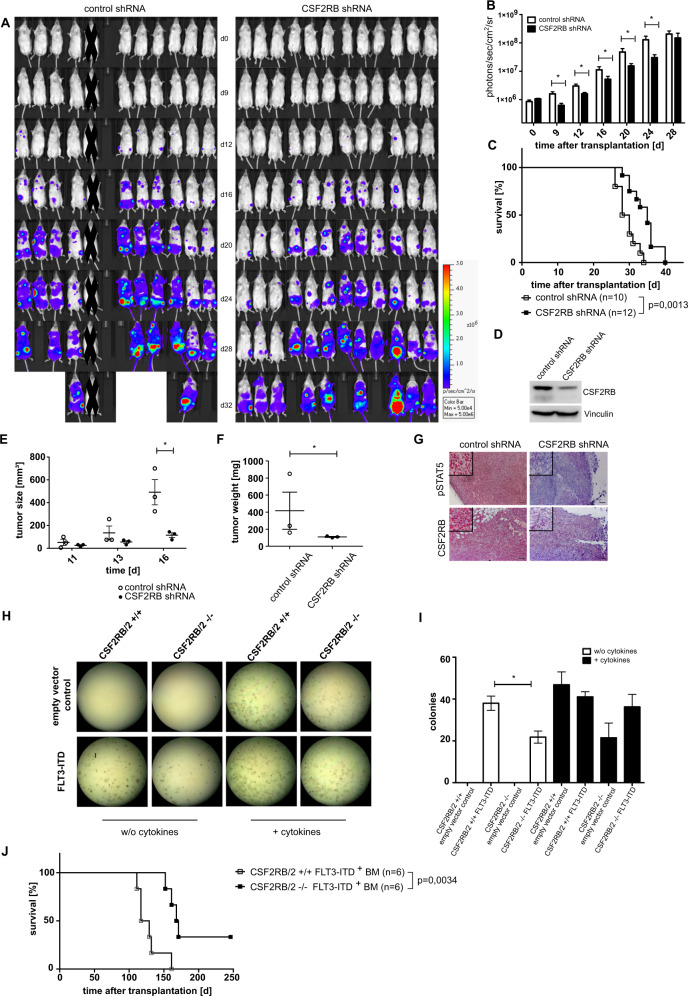
Loss of CSF2RB attenuates FLT3-ITD-dependent transformation and proliferation and prolongs survival in xenograft and bone marrow transplantation models. **A**–**D** Immunodeficient *Rag2/Il2rg-*mutant mice were sublethally irradiated and injected intravenously with FLT3-ITD-positive MOLM-13 cells stably expressing CSF2RB shRNA (*n* = 12) or control shRNA (*n* = 10) together with luciferase. One animal was excluded as no signs of engraftment could be detected at any time point. **A** Mice were injected intraperitoneally with luciferin and subjected to bioluminescence imaging, as indicated. **B** Quantification of bioluminescence is shown. *P* value was calculated using a two-sided Mann–Whitney test. **C** Survival curve of mice transplanted with MOLM-13 cells expressing CSF2RB shRNA or control shRNA. *P* values were calculated by Mantel–Cox test, *n* represents biologically independent mice. **D** The same MOLM-13 cells were subjected to lysis, SDS–PAGE, and western blot using the indicated antibodies. **E**–**G** FLT3-ITD positive MOLM-13 cells expressing CSF2RB shRNA or control shRNA were injected subcutaneously into the flank (*n* = 3 for both groups) of immunodeficient *Rag2/Il2rg-*mutant mice. **E** Tumor size was measured at given days by caliper. **F** Tumor weight was measured after sacrificing the animals on day 17 (*p* value of 0.0014 calculated by *F* test). **G** Sectioned tumors were fixed in 4% paraformaldehyde and then subject to immunohistochemical staining (by labeled Streptavidin–Biotin method) with the indicated antibodies, using hematoxylin for counterstaining. Representative images are shown (Axio Imager.M2; EC Plan-Neofluar ×10/0.3), scale bar = 100 µm. **H**, **I** Bone marrow of *Csf2rb/Csf2rb2* double-knockout mice and wildtype littermates was transduced with FLT3-ITD or empty vector with GFP as transduction control. In all, 3000 GFP-positive sorted cells were cultured in methylcellulose ± cytokines (SCF, IL-3, IL-6, EPO, insulin, transferrin, iron-saturated). Representative images of one experiment (**H**) and analysis of three independent experiments (**I**) are shown. **J** Lethally radiated C57/BL6 littermates were transplanted with bone marrow from *Csf2rb/Csf2rb2* double knockout or wildtype littermates transduced with FLT3-ITD 598/599[12] construct with GFP as transduction control; non-transduced bone marrow was supplemented to equalize the number of GFP-positive cells (*n* = 6 for both groups). The recipients’ survival is shown, with day 246 after transplantation as an endpoint. *P* value was calculated by Mantel–Cox test; *n* represents biologically independent mice.

We next examined the role of CSF2RB in vivo using two different approaches. First, we performed xenografts using immunodeficient *Rag2/Il2rg-*mutant mice as transplant hosts for MOLM-13 cells. Mice were sublethally irradiated and MOLM-13 cells expressing CSF2RB shRNA or control shRNA were transplanted via tail vein injection. The expression of a luciferase reporter served for visualization of leukemic cell expansion after engraftment. Consistent with our in vitro findings, MOLM-13 cells expressing CSF2RB shRNA showed significantly less expansion in vivo (Fig. 3A, B). Moreover, mice transplanted with CSF2RB knockdown cells showed significantly longer survival compared to littermates transplanted with MOLM-13 cells expressing control shRNA (Fig. 3C). CSF2RB knockdown efficiency was confirmed by western blot analysis prior to transplantation (Fig. 3D). Similar results were obtained in a subcutaneous tumor model. MOLM-13 cells expressing either CSF2RB shRNA or control shRNA were injected subcutaneously into the flanks of *Rag2/Il2rg-*mutant mice and tumor growth was monitored by caliper measurements. MOLM-13 tumors with diminished expression of CSF2RB showed slower growth rates (Fig. 3E, F). Immunohistological analysis of paraffin-embedded tumors revealed almost absent phosphorylation of STAT5 in CSF2RB diminished tumor material. In contrast, control shRNA tumor samples showed uniform and strong phosphorylation of STAT5 (Fig. 3G).

Second, we used a CSF2RB knockout mouse strain. In contrast to the human receptor system, mice express a second IL-3-specific beta subunit (CSF2RB2 or BIL3), which forms a high-affinity IL-3-binding complex with IL3RA. For this reason, we used mice homozygous for both knockout genes (*Csf2rb* and *Csf2rb2*). First, we analyzed the ability of *Csf2rb/Csf2rb2* double-knockout BM transduced with MIG FLT3-ITD to form colonies in methylcellulose assays. BM derived from *Csf2rb/Csf2rb2* double-knockout mice yielded a lower transformation capacity compared to BM derived from WT littermates (Fig. 3H, I). In a BM transplantation model, we transplanted BM derived from C57BL/6 J *Csf2rb/Csf2rb2* double-knockout mice and WT littermates, both transfected with FLT3-ITD, into irradiated C57BL/6 J WT recipient mice. As described previously, recipient animals developed a T-cell malignancy (data not shown). Recipients of *Csf2rb/Csf2rb2* double-knockout mice BM showed significantly prolonged survival compared to recipients of WT BM (Fig. 3J). All six animals transplanted with FLT3-ITD transfected WT BM succumbed to the disease. Two out of six recipients of FLT3-ITD transfected CSF2RB knockout BM did not develop a disease. GFP positivity of BM cells confirmed expression of FLT3-ITD and expansion preferentially in animals that received CSF2RB WT BM. (Suppl. Fig. 4A). Together, these experiments demonstrate the biologic relevance of CSF2RB in mediating FLT3-ITD-dependent transforming capacity and oncogenic potential in vivo.

### Activation of CSF2RB by FLT3-ITD does not require complex formation with IL3RA

**Fig. 4:**
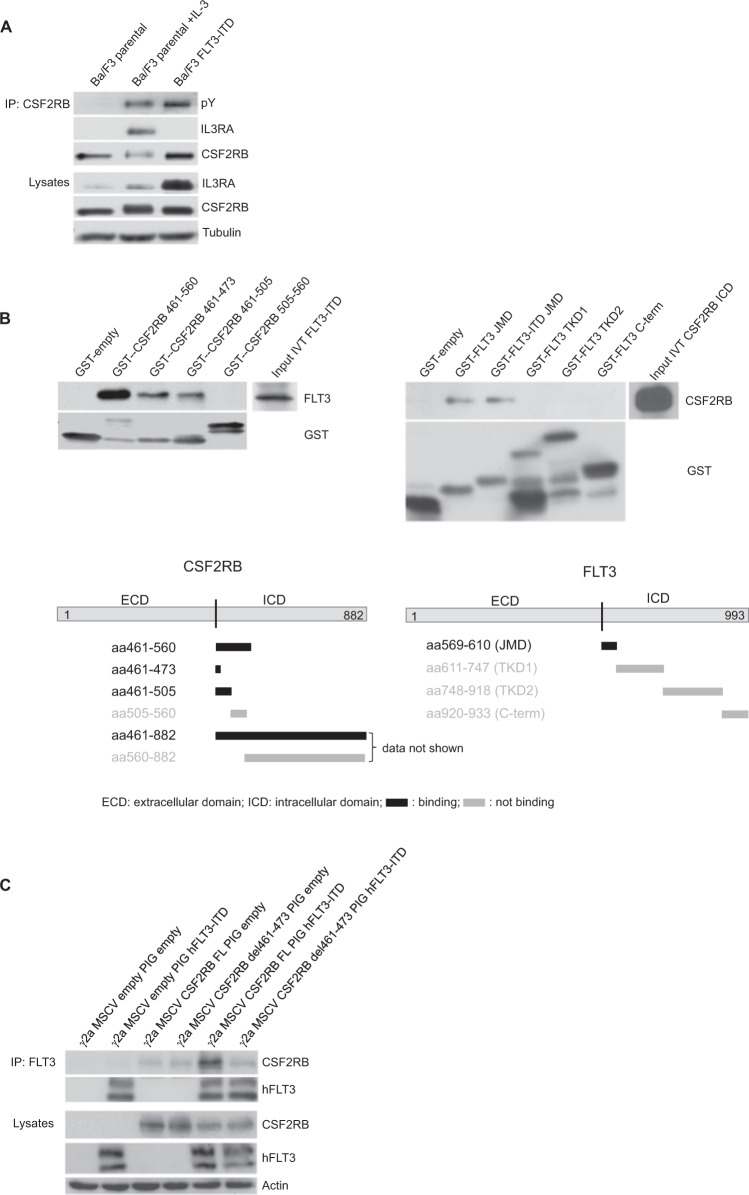
Interaction of CSF2RB and FLT3 is independent of physiological receptor complex formation and takes place at the cytosolic site close to the membrane. **A** Parental Ba/F3 cells and FLT3-ITD-expressing Ba/F3 cells were serum-deprived for 4 h and left untreated or incubated with IL-3 at 2 ng/ml. Whole-cell lysates were subjected to immunoprecipitation using antibodies against CSF2RB. Immunoprecipitates and whole-cell lysates were subjected to SDS–PAGE and western blot analysis using indicated antibodies. **B** Mapping of the CSF2RB and FLT3 binding sites by GST-pulldown assay. Schematic diagrams show the regions used for glutathione S-transferase tagged peptides captured by glutathione-coated agarose beads. In vitro translated intracellular domains of FLT3-ITD and CSF2RB served as binding partners (input). Incubation was performed overnight; interaction complexes were separated by SDS–PAGE and subjected to western blot analysis using the indicated antibodies. *JMD* juxtamembrane domain, *TKD1* tyrosine kinase domain 1, TKD2 tyrosine kinase domain 2. **C** CSF2RB-deficient gamma-2A cells were transduced with two different variants of CSF2RB (full-length (FL) or del461-473) and human FLT3-ITD or in combination with empty vectors, as indicated. Whole-cell lysates were subjected to immunoprecipitation using antibodies against FLT3. Immunoprecipitates and whole-cell lysates were subjected to SDS–PAGE and western blot analysis using indicated antibodies.

As canonical activation of CSF2RB requires extracellular binding of a particular cytokine to the corresponding alpha subunit and formation of a dodecamer complex [29], we investigated whether FLT3-ITD induced phosphorylation of CSF2RB is dependent on complex formation with IL3RA. In FLT3-ITD expressing cells, we did not observe the formation of a CSF2RB/IL3RA complex, although CSF2RB was phosphorylated. By contrast, a CSF2RB/ILR3RA complex was detected in parental Ba/F3 cells stimulated with IL-3 (Fig. 4A). Thus, we conclude that the interaction of CSF2RB and FLT3-ITD along with further downstream signaling events does not require canonical receptor complex formation.

### Interacting regions of CSF2RB and FLT3 are located membrane-proximal

To determine the interacting domains of the CSF2RB/FLT3 complex, we performed GST-pulldown mapping experiments. We used GST-tagged peptide fragments representing different intracellular regions of each receptor and incubated purified fragments with in vitro translated cytoplasmic domains of the interacting receptor. Binding was assessed by immunoblotting. We identified a CSF2RB membrane-proximal region of 12 amino acids (aa461–473) to be necessary and sufficient to bind FLT3 (Fig. 4B, left panels). Of note, this region overlaps with the known interacting regions of CSF2RB for JAK2 (aa 458–95) and Lyn (aa 457–65) [30–32]. Consistent with these results, the reconstituted full-length CSF2RB, but not CSF2RB del461-473 established binding to FLT3-ITD in CSF2RB-deficient human fibrosarcoma Gamma-2A cells (Fig. 4C). Within FLT3, a 42 amino-acid peptide containing the JMD was identified as CSF2RB-binding domain (Fig. 4B, right panels). Both FLT3-JMD and FLT3-ITD-JMD retained CSF2RB binding.

### Phosphorylation of CSF2RB by FLT3-ITD requires only one phosphorylated tyrosine within the JMD

**Fig. 5:**
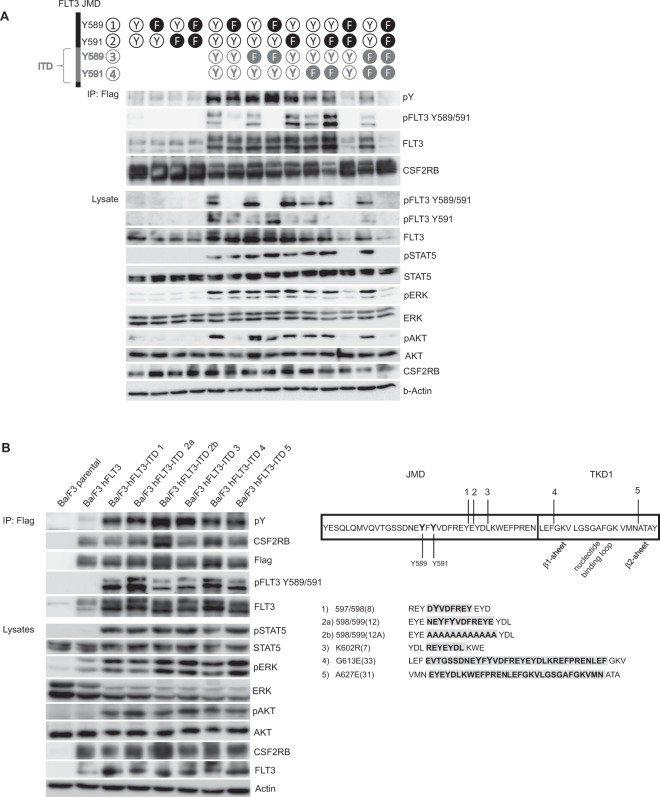
Phosphorylation of CSF2RB by FLT3-ITD requires only one phosphorylated tyrosine within the JMD and takes place regardless of ITD sequence and insertion site. **A** Ba/F3 cells were transduced with CSF2RB-Flag and either FLT3 or FLT3-ITD 598/599 [12] containing mutations of tyrosines 589 and 591 to phenylalanine as indicated. Cells were serum-deprived for 5 h and co-immunoprecipitations were performed using anti-flag beads capturing CSF2RB. Immunoprecipitates and whole-cell lysates were subjected to SDS–PAGE and western blot analysis using indicated antibodies. **B** Parental Ba/F3 cells and Ba/F3 cells expressing CSF2RB-Flag in combination with either FLT3 or one FLT3-ITD variant were serum-deprived for 5 h. Co-immunoprecipitations were performed using anti-flag beads capturing CSF2RB. Immunoprecipitates and whole-cell lysates were subjected to SDS–PAGE and western blot analysis using indicated antibodies. Sequences and insertion sites of utilized FLT3-ITD variants are depicted on the right.

The JMD of FLT3 contains two tyrosines 589 and 591 that are autophosphorylated in FLT3-ITD and are required for FLT3-ITD mediated phosphorylation of STAT5 (Fig. 1A) [33]. These two tyrosines are not phosphorylated after stimulation with FLT-ligand or in FLT3-ITD in the presence of TKI (Fig. 1A), indicating their significance for FLT3-ITD-mediated phosphorylation of CSF2RB. We analyzed known FLT3-ITD sequences (https://cancer.sanger.ac.uk/cosmic) for the presence of tyrosines 589 and 591. Frequently, one or both of the tyrosines 589 and 591 are part of the duplicated sequence. We aimed to explore the significance of tyrosines 589 and 591 within the ITD for FLT3-ITD autophosphorylation and consecutive CSF2RB phosphorylation. For this purpose, we mutated one or both tyrosines to phenylalanine either within the original position of the JMD or within the individual tandem duplication of a given FLT3-ITD variant, or both. For this, we chose ITD variant 598/599 [12], involving duplication of aa587–598, as the ITD is located within the JMD and contains both relevant tyrosines [34]. Surprisingly, either one of the tyrosines in the original position of the JMD alone was sufficient for autophosphorylation of FLT3-ITD and consecutive CSFR2B phosphorylation (Fig. 5A). Interestingly, when both tyrosines in the JMD were simultaneously mutated to phenylalanine, the tyrosines present in the tandem duplication were not able to reconstitute phosphorylation of FLT3 and CSF2RB. In conclusion, this experiment demonstrates that the tyrosines 589 and 591 in the original position of the JMD are necessary and sufficient for autophosphorylation of FLT3-ITD and consecutive CSF2RB phosphorylation, and that additional tyrosines within the ITD sequence are not required for downstream signaling or phosphorylation of CSF2RB.

### The role of the ITD insertion sequence or site for CSF2RB activation

We next asked whether the inserted ITD sequence or the localization of the ITD is critical for FLT3 downstream signaling or CSF2RB phosphorylation. Most FLT3-ITD variants occur within the JMD whereas ITDs in the tyrosine kinase domain are rare [35, 36]. We selected three different ITDs, which are located within the JMD [34, 37], and two ITDs, which are located within the tyrosine kinase domain 1 (TKD1), one within the beta1-sheet [34] and one within the beta2-sheet [38] (Fig. 5B). We performed co-immunoprecipitations with Ba/F3 cells expressing FLAG-tagged human CSF2RB together with different FLT3-ITD variants. Compared with WT control, all ITDs bound CSF2RB, led to CSF2RB phosphorylation and STAT5 activation (Fig. 5B), and rendered Ba/F3 cells IL-3 independent (data not shown). We then replaced aa587–598 of ITD 598/599 [12] by alanines [34]. Interestingly, this artificial solely alanine containing ITD still retained transformation (data not shown) and signaling competence indicating that the ITD insertion site and sequence are not critical for CSF2RB phosphorylation, STAT5 activation, and transformation (Fig. 5B).

Taken together our findings demonstrate that CSF2RB directly interacts with FLT3 and is phosphorylated in an FLT3-ITD-dependent fashion. It participates in FLT3-ITD induced activation of STAT5 and plays a pivotal role in promoting the oncogenic potential of FLT3-ITD in vitro and in vivo.

## Discussion

In our previous work, we demonstrated that activating mutations in JAK1, 2, and 3 mediate resistance to FLT3 inhibitor treatment in FLT3-mutated AML and lead to reactivation of CSF2RB [19]. We here show for the first time that CSF2RB directly interacts with and is phosphorylated by FLT3-ITD in human AML cell lines and in primary AML blasts expressing FLT3-ITD. CSF2RB activation leads to STAT5 activation [17, 18], the key oncogenic signal of FLT3-ITD [3–5], and we were able to show that CSF2RB knockdown attenuated FLT3-ITD dependent STAT5 activation and cell growth. These observations demonstrate that CSF2RB constitutes a key element in FLT3-ITD-oncogenic signaling. To support this, we demonstrated that CSF2RB knockdown decreased proliferation in human AML cell lines expressing FLT3-ITD and in xenograft experiments, led to reduced disease burden and increased survival. Finally, BM from CSF2RB knockout mice transfected with FLT3-ITD demonstrated decreased colony formation capacity and transplanted into lethally irradiated mice, delayed disease onset, and increased survival. In addition, CSF2RB knockdown sensitized FLT3-ITD expressing cell lines to FLT3 inhibition at inhibitor concentrations achievable in plasma of treated patients [39, 40]. These results demonstrate the biological significance of CSF2RB in FLT3-ITD-positive AML. IL3RA and CSF2RB are preferentially expressed in FLT3-mutated AML [41]. Overexpression of IL3RA in AML is associated with poor prognosis [42]. IL3RA is under investigation as a treatment target employing antibody and CAR strategies [43–45]. Our results suggest that therapeutic use of peptides blocking the CSF2RB/FLT3-ITD-binding interface might be used to synergize with therapeutic FLT3 kinase inhibitors to improve response and attenuate the emergence of resistance in FLT3-mutated AML.

STAT5 is a critical mediator of myeloid transformation by FLT3-ITD [6]. The mechanism of FLT3-ITD-dependent STAT5 phosphorylation in AML is still unclear. Direct activation by FLT3-ITD has been suggested [6]. In our experiments, the impaired oncogenic potential of FLT3-ITD upon CSF2RB knockdown was associated with reduced STAT5 phosphorylation, indicating that signaling via CSF2RB contributes to FLT3-ITD dependent STAT5 activation. In accordance, Riccioni et al. [41] previously correlated high expression of CSF2RB in FLT3-ITD positive AML patients with stronger STAT5 phosphorylation. In addition to CSF2RB, FLT3-ITD might also bind and activate other receptors and downstream mediators to contribute to STAT5 activation and FLT3-ITD-dependent downstream signaling. In line with this possibility, it has been demonstrated that activation of signaling intermediates like GRB2, SRC, BTK, and SYK, which are all involved in receptor signaling, contributes to FLT3-ITD-dependent STAT5 activation [7–11]. Although the interaction of CSF2RB and FLT3-ITD has not been described previously, the interaction between CSF2RB and EPOR and KIT receptors has been reported [46, 47]. Future experiments will elucidate the cross-talk between FLT3-ITD and other receptors.

To better understand the mechanism of FLT3-ITD-dependent CSF2RB activation, we performed interaction mapping experiments. CSF2RB activation required the presence of FLT3-ITD. FLT3 also interacted with but failed to activate CSF2RB, even after stimulation with FLT-ligand. Of note, although our in vitro studies did not demonstrate a difference between FLT3 and FLT3-ITD in CSF2RB binding, we detected less CSF2RB/FLT3 than CSF2RB/FLT3-ITD interactions in our PLA experiments suggesting that within a membrane structure of cells, the differential conformation of FLT3-ITD might favor interaction with CSF2RB. Inhibition of FLT3-ITD kinase activity did not interfere with the interaction but abrogated CSF2RB phosphorylation. In addition, the interaction of CSF2RB with the corresponding alpha chain [29] was not required for CSF2RB activation by FLT3-ITD. Thus, the interaction of CSF2RB with autophosphorylated FLT3-ITD is necessary for CSF2RB activation. We mapped the interaction site of both proteins to a membrane-proximal region of both receptors, which in the future might allow designing therapeutic peptides that interfere with FLT3-ITD dependent CSF2RB activation.

We could demonstrate that phosphorylation of at least one of the two tyrosines 598 and 591 within the JMD of FLT3-ITD is required for phosphorylation of CSF2RB, and that neither the sequence nor the insertion site of the ITD affects the ability to activate CSF2RB. In addition, we demonstrated that only tyrosine 599 is phosphorylated in FLT3 expressing cells after treatment with FLT-ligand, whereas phosphorylation of tyrosines 589 and 591 was restricted to FLT3-ITD-expressing cells and thus constitutes a pathological feature of FLT3-ITD. Our observations are in agreement with a working model of FLT3 activation by ITDs proposed by Chan et al. [48]. According to this model, the elongation of the JMD of FLT3 via ITDs allows the tyrosines 598 and 591 to gain access to the catalytic aspartate at codon 811 allowing their phosphorylation, a conformational change to cis, and autophosphorylation of the kinase domain of FLT3, which then leads to further downstream signaling [49]. In this line, the elongation caused by ITD insertions might bring the two tyrosines 598 and 591 close enough to FLT3’s own catalytic aspartate, but also to critical sites within CSF2RB, allowing their processive phosphorylation. The differential competence of various ITDs to bind and activate signal mediators might explain the prognostic difference of specific FLT3-ITD mutations in AML, and might also explain the superior prognosis of FLT3-TKD [50, 51]. In addition, through the elongation of FLT3 adjacent to the membrane and the more open accessibility of tyrosines 589 and 591, other adaptor proteins might get activated and mediate the phosphorylation of CSF2RB.

We here demonstrate the pivotal role of CSF2RB in FLT3-ITD oncogenic signaling and cellular transformation in vitro and in vivo, suggesting it to be a rational drug target in FLT3-ITD positive AML.

## Supplementary information

Supplementary
