# Supplementary material for: The IL-3, IL-5, and GM-CSF common receptor beta chain mediates oncogenic activity of FLT3-ITD-positive AML
Source: Leukemia. 2021 Nov 8;36(3):701–11. doi: 10.1038/s41375-021-01462-4 (PMC8885422; doi:10.1038/s41375-021-01462-4)
Supplement: Supplementary file 1 — Supplementary [file 41375_2021_1462_MOESM1_ESM.pdf]

Supplementary Figure 1

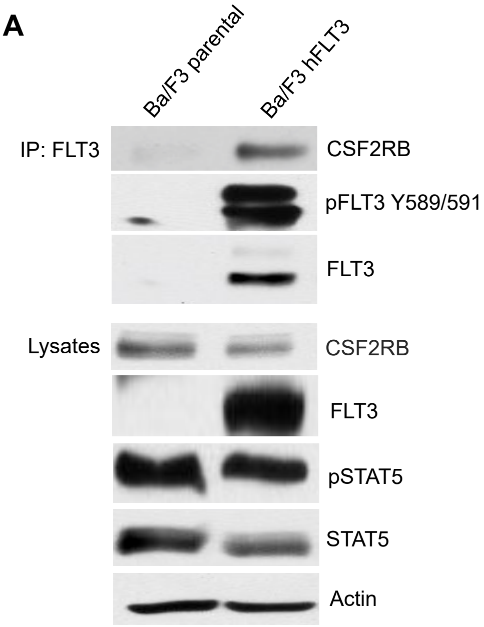

Supplementary Figure 2

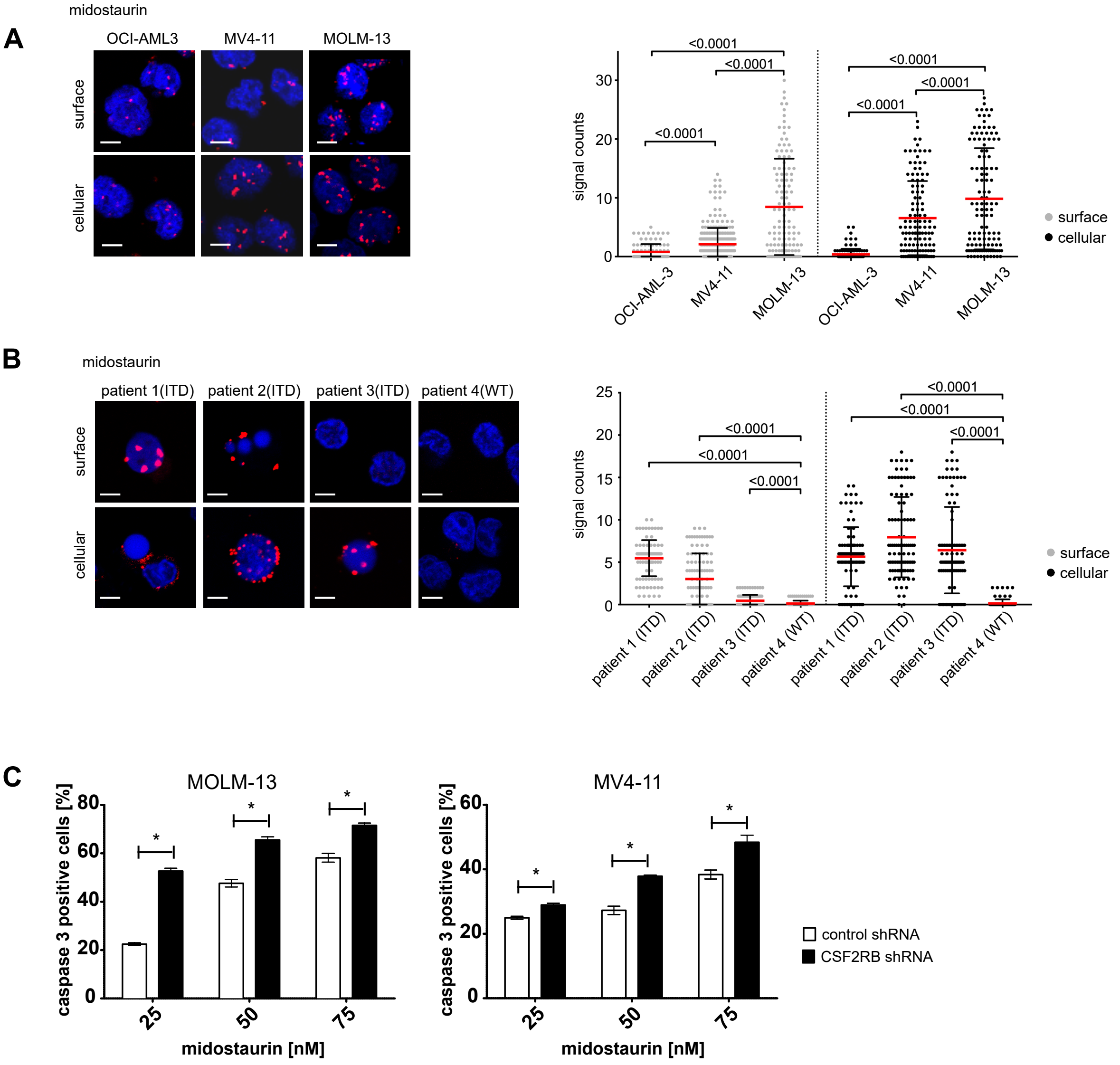

Supplementary Figure 3

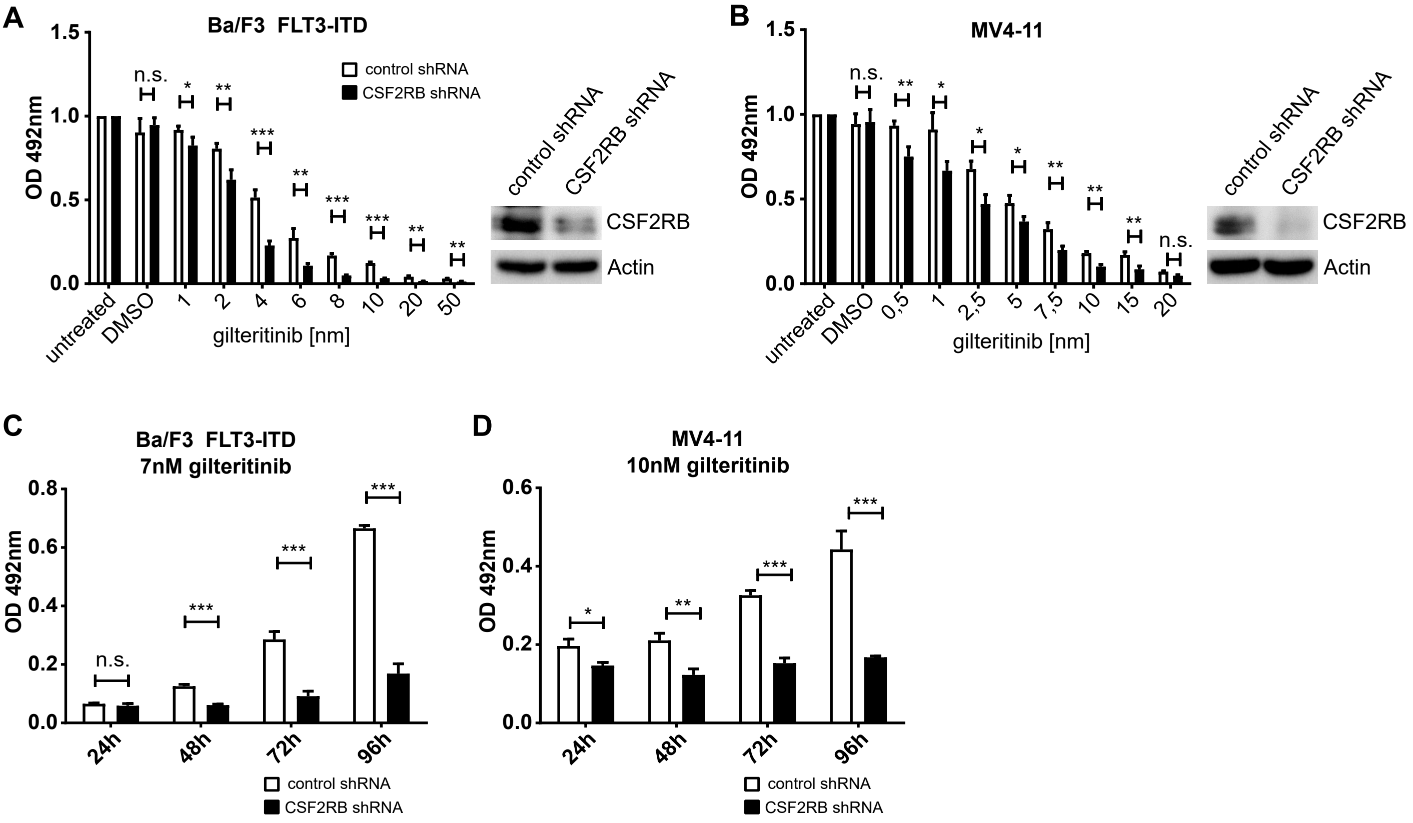

Supplementary Figure 4

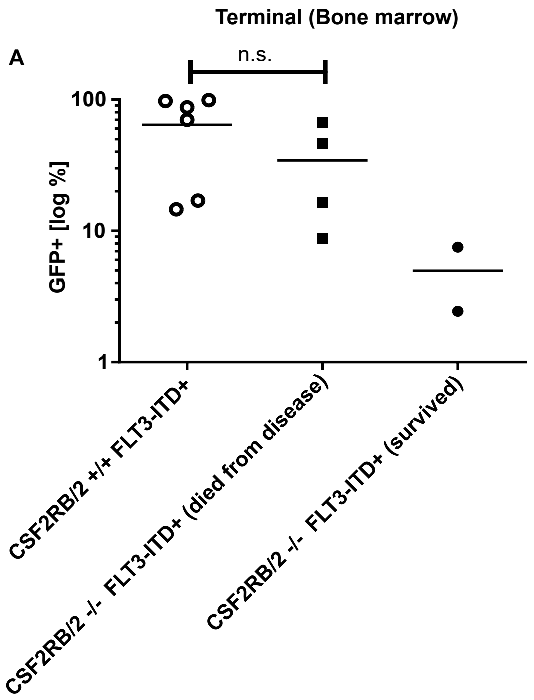

Supplementary Figure 1: Endogenous CSF2RB interacts with FLT3. (A) Ba/F3 cells expressing FLT3-ITD were serum deprived for 5h. Co-immunoprecipitation was performed using FLT3-antibody. Immunoprecipitates and whole cell lysates were subjected to SDS-PAGE and western blot analysis using indicated antibodies.

Supplementary Figure 2: Inhibition of FLT3 via midostaurin leaves the interaction of CSF2RB and FLT3 unaffected whereas it increases caspase activity in CSF2RB diminished cells. (A) Proximity ligation assay (1-PLA) was performed using oligo-coupled primary antibodies against FLT3 and CSF2RB. FLT3 expressing OCI-AML3 cells and FLT3-ITD expressing MV4-11 and MOLM-13 cells were incubated with 100nM midostaurin for 1h and subsequently fixed (surface) or fixed and permeabilized (cellular) prior to PLA reaction. Red dots indicate the occurrence of a close FLT3: CSF2RB proximity. Nuclei were counterstained with DAPI. Representative images are shown (Zeiss 780 Meta confocal microscope; Objektiv NA 1.4), scale bar= 10µm. Quantification of the PLA signals is shown right, p <0,0001. (B) Blast cells from three FLT3-ITD positive AML patients and one FLT3-ITD negative AML patient (patient 4) were isolated from peripheral blood using Ficoll density gradient. Cells were incubated with 100nM midostaurin for 1h, fixed (surface) or fixed and permeabilized (cellular) and PLA was performed (as described in A). Red dots indicate the occurrence of a close FLT3: CSF2RB proximity. Nuclei were counterstained with DAPI. Representative images are shown (Zeiss 780 Meta confocal microscope; Objective NA 1.4), scale bar= 10µm. Quantification of the PLA signals is shown right, p <0,0001. (C) FLT3-ITD positive AML cell lines MOLM-13 and MV4-11 were transduced with inducible CSF2RB shRNA or control shRNA respectively and cultured in the presence of doxycycline for at least 48h prior to experiment and midostaurin at the indicated concentrations. Cells were fixed and permeabilized and stained with anti-Active Caspase-3. Subsequently, flow cytometry was performed to analyze percentage of Caspase-3 positive cells.

Supplementary Figure 3: CSF2RB sensitizes cells to FLT3 inhibition with gilteritinib. (A-D) Ba/F3 cells transduced with FLT3-ITD and FLT3-ITD positive AML cell line MV4-11 were transduced with inducible CSF2RB shRNA or control shRNA respectively and cultured in the presence of doxycycline for at least 48h prior to and during experiments. (A, B) Cell viability was determined using MTS-assay. Cells were seeded in a density of 6000 cells per well in 96-well plates and cultured in the presence of gilteritinib at the indicated concentrations. Proliferation was measured in triplicates as formazan absorption after 72h at 490nM. Cell lysates were subjected to SDS-PAGE and western blot analysis using indicated antibodies to

confirm knockdown. (C, D) Cells were seeded in a density of 6000 cells per well in 96-well plates and cultured in the presence of gilteritinib at the indicated concentrations. Cell viability was measured daily by formazan absorption at 490nm.

Supplementary Figure 4: GFP positivity of transplanted mice in Figure 3J. (A) After sacrificing the mice, the bone marrow of the animals was harvested and evaluated for GFP+ cells by FACS. In the *Csf2rb/Csf2rb2* double knockout group, the non-diseased animals are presented separately. Each data point shows on individual animal.
